# The β-catenin-LINC00183-miR-371b-5p-Smad2/LEF1 axis promotes adult T-cell lymphoblastic lymphoma progression and chemoresistance

**DOI:** 10.1186/s13046-023-02670-9

**Published:** 2023-04-28

**Authors:** Wei-Juan Huang, Song-Bin Guo, Hui Shi, Xin-Ling Li, Yong Zhu, Mei Li, Li-Yan Song, Rong-Min Yu, Qing-Qing Cai, Xiao-Peng Tian

**Affiliations:** 1 Department of Medical Oncology, Sun Yat-sen University Cancer Center, Guangzhou, China; 2 State Key Laboratory of Oncology in South China, Collaborative Innovation Center of Cancer Medicine, Sun Yat-sen University Cancer Center, Guangzhou, China; 3 Department of Pharmacology, College of Pharmacy, Jinan University, Guangzhou, China; 4 Biotechnological Institute of Chinese Materia Medical, Jinan University, Guangzhou, China; 5 Department of General Surgery, The First Affiliated Hospital of Anhui Medical University, Hefei, China; 6 Anhui Public Health Clinical Center, Hefei, China; 7 Department of Pathology, Sun Yat-sen University Cancer Center, Guangzhou, China

**Keywords:** T-LBL, Chemoresistance, LINC00183, miR-371b-5p, Smad2, LEF1

## Abstract

**Background:**

High-intensity chemotherapy regimens are often used in adult T-cell lymphoblastic lymphoma (T-LBL) patients. Nevertheless, the response rate remains unsatisfactory due to emergence of chemoresistance. Growing evidence has shown that long non-coding RNAs (lncRNAs) are involved in tumor progression and chemoresistance. Herein, we investigated the potential role of lncRNAs in T-LBLs.

**Methods:**

RNAseq was used to screen and identify candidate lncRNAs associated with T-LBL progression and chemoresistance. Luciferase reporter assay was used to examine the binding of miR-371b-5p to the 3’UTR of *Smad2* and *LEF1*, and the binding of TCF-4/LEF1 to the promoter of LINC00183. Chromatin immunoprecipitation assay was undertaken to analyze the connection between LEF1 and the LINC00183 promoter region. RNA immunoprecipitation assays were used to explore the mechanism whereby LINC00183 regulated miR-371b-5p. MTT and flow cytometry assays were used to measure apoptosis of T-LBL cells.

**Results:**

LINC00183 was upregulated in T-LBL progression and chemoresistant tissues in both the Sun Yat-sen University Cancer Center dataset and the First Affiliated Hospital of Anhui Medical University dataset. High expression of LINC00183 was correlated with poorer overall survival and progression-free survival of T-LBL patients compared to those with low expression of LINC00183. Furthermore, miR-371b-5p was negatively regulated by LINC00183. In vivo and in vitro assays showed that LINC00183-mediated T-LBL chemoresistance depended on miR-371b-5p expression. The direct binding of miR-371b-5p to Smad2 and LEF1 was verified by luciferase assays. It was shown that TCF4/LEF1 could bind to the LINC00183 promoter site and increase its transcript level. Downregulation of miR-371b-5p led to increased expression of Smad2/LEF1, and in turn increased LINC00183 expression. Additionally, phospho-Smad2 promotes nuclear translocation of β-catenin, LINC00183 downregulation decreased chemoresistance induced by β-catenin and TGF-β1 in T-LBL cells.

**Conclusion:**

We unraveled a β-catenin-LINC00183-miR-371b-5p-Smad2/LEF1 feedback loop that promotes T-LBL progression and chemoresistance, indicating that LINC00183 may serve as a potential therapeutic target in T-LBLs.

**Supplementary Information:**

The online version contains supplementary material available at 10.1186/s13046-023-02670-9.

## Background

T-cell lymphoblastic lymphoma (T-LBL), an aggressive non-Hodgkin’s lymphoma, arises from precursor T lymphoblasts and primarily occurs in adolescents and young adults [1–3]. Though T-LBL is grouped together with T cell acute lymphoblastic leukemia (T-ALL) per current WHO classification, the two entities are notably different in genetic profile, clinical presentation, group stratification, and prognosis [4–6]. Intensive chemotherapy has greatly improved the survival of T-LBL patients; however, the 3-year disease-free survival (DFS) rate remains to be 75-90% [7–10]. Additionally, after initial complete remission (CR), about 40% of adult patients would relapse. The prognosis is poor for T-LBL patients who fail to achieve CR or relapse after CR [11]. Drug resistance is mostly responsible for CR failure or post-CR relapse [12]. Therefore, it is crucial to comprehend the mechanisms underlying T-LBL chemoresistance so as to develop novel potent therapeutic approaches.

Non-coding RNAs (ncRNAs) are implicated in a number of pathophysiological processes, particularly in cancer progression [13, 14]. Though the roles and mechanisms of microRNAs (miRNAs) have been extensively studied in human cancer, the functions of long non-coding RNAs (lncRNAs) remain to be fully elucidated [15, 16]. Genes are regulated by lncRNAs through a variety of manners, but it is still challenging to identify cancer-related lncRNAs. There are numerous theories to explain how lncRNAs work, one of which is the competitive endogenous RNA (ceRNA) hypothesis [17, 18]. According to this theory, lncRNAs harbor miRNA-response elements (MREs), which compete with miRNAs for binding and counteract their inhibitory effects on target mRNAs. It has been validated that “lncRNA-miRNA-mRNA” network exists and matters in numerous types of cancers. Meanwhile, it is still unclear how these networks work in T-LBLs [19, 20].

Currently, lncRNAs have been shown to play an important role in chemotherapeutic drug resistance, tumor invasion and metastasis and other biological functions. However, there are few studies on the function of lncRNAs in T-LBLs [21]. The activation of Wnt/β-catenin signaling plays a critical role in T-LBL development [22]. Phospho-Smad2/3 has been reported to promote the stability and nuclear translocation of β-catenin [23]. However, few studies have shown that lncRNAs mediate the interaction between the Wnt/β-catenin signaling pathway and the TGF-β1/Smad pathway [24]. In this study, we found that LINC00183 was upregulated in T-LBL progression and chemoresistant tissues. We have shown that the LINC00183-miR-371b-5p-Smad2/Lymphoid enhancer-binding factor 1 (LEF1) axis promotes T-LBL drug resistance. Notably, the Wnt/β-catenin and TGF-β1/Smad signaling pathway may also be a regulator of LINC00183. The β-catenin/LEF1-LINC00183-miR-371b-5p-Smad2/LEF1 feedback loop may offer novel insights into T-LBL drug resistance and LINC00183 could serve as a novel potential therapeutic target.

## Materials and methods

### Cells

T-LBL cell lines Jurkat and SUP-T1 (American Type Culture Collection, VA, USA) were maintained in RPMI-1640 (Gibco, USA) supplemented with 10% fetal calf serum (Gibco) at 37℃ in a humidified incubator equilibrated with 5% CO_2_.

### Patients and tissue specimen acquisition

Formalin-fixed, paraffin-embedded (FFPE) tissues were obtained from 12 adult T-LBL patients, including 3 chemoresistant patients who failed to achieve CR after induction chemotherapy, 3 treatment-sensitive patients who achieved CR after two cycles of induction chemotherapy, 3 patients who achieved CR after induction chemotherapy but relapsed within 6 months, and 3 patients who achieved CR after induction chemotherapy and did not relapse within 3 years. The patients received treatment at Sun Yat-sen University Cancer Center (SYSUCC, Guangzhou, China) between January 1st, 2014 and December 30th, 2017. Another cohort study consisted of T-LBL FFPE samples from 92 adult (18–65 years) patients taking the BFM or hyper-CVAD regimen in the first-line setting at SYSUCC, and 39 adult patients who were treated at The First Affiliated Hospital of Anhui Medical University (AHAMU, Hefei, China) between January 1st, 2010 and December 30th, 2017. The patients’ information was provided in the Table S1.

T-LBL was diagnosed per the 2016 WHO criteria and confirmed by two independent physicians (Mei Li and Yong Zhu) [25]. The immunophenotype required for T-LBL diagnosis is TdT(+), cCD3(+), CD1α(±), CD2(±), CD4(±), CD5(±), CD7(±) and CD8(±). Induction was done with vincristine, daunorubicin, *L*-asparaginase, prednisone, and cyclophosphamide, cytarabine, and 6-mercaptopurine (VDLP-CAM) in the BFM regimen, or 2–4 cycles of hyper-CVAD. The clinical specimens were collected via biopsy or resection, and later preserved and paraffin-embedded. The samples from the SYSUCC were analyzed by RNA sequencing to identify differentially expressed lncRNAs. Positron emission tomography computed tomography (PET-CT), CT, and/or bone marrow biopsy were required for confirmation of CR and relapse per the Cheson criteria [26].

All clinical samples were obtained with informed consent of the patients in accordance with the Institutional Review Board-approved path. This study was authorized by the Institute Research Ethics Committee of SYSUCC.

### RNA extraction, purification and sequencing

Total cellular RNA was isolated from FFPE tissues using the RecoverALL Total Nucleic Acid Isolation kit (Cat #AM1975, Invitrogen, Carlsbad, CA, USA) following the manufacturer’s instructions. RNA integrity was checked using Agilent 2100 Bioanalyzer (Agilent technologies, CA, USA) and RNA was digested with RNase-Free DNase (NEB, MA, USA), and then purified using the RNAClean XP Kit (Cat#A63987, Beckman Coulter, CA, USA) and quantified using NanoDrop 2000 (Thermo Fisher Scientific, MA, USA). The purified total RNA was fragmented, followed by first strand cDNA synthesis, second strand cDNA synthesis, cDNA ligation, rRNA removal, and amplification to complete the sequencing library construction. The library was built using Qubit® 2.0 Fluorometer for concentration determination and Agilent 4200 for size detection. NOVA 6000 sequencer was used and PE150 mode was selected for sequencing.

### MTT assays

Cell viabilities were studied using MTT assays (Sigma, MO, USA) as instructed by the manufacturer. Cells were seeded at a density of 3000 cells per well in 96-well plates with or without 100 ng/mL doxorubicin (Sigma-Aldrich). After 12 to 48 h, cell viability was detected. Two hundred µL of MTT solution was added to each well 48 h post treatment, and after incubation for 4 h, and the optical density was read at 490 nm using a microplate reader [27]. All experiments were performed independently at least in triplicates.

### Flow cytometry

Cells were cultured for 36 h followed by treatment with 100 ng/ml doxorubicin for 24 h and then stained with propidium iodide and annexin V-APC. Apoptotic cells were detected using cytoFLEX (Beckman Coulter, CA, USA) following the manufacturer’s suggestion (BioVision, CA, USA).

### Cell fractionation assays

Nucleic and cytoplasmic RNA were isolated using the PARIS Kit (Life Technologies, CA, USA) according to the manufacturer’s instruction. Small nuclear RNA U2 (snRNA U2) and actin were taken as positive references for the nucleic and the cytoplasmic fraction, respectively.

### Quantitative real-time PCR

Total RNA was extracted using TRIzol reagent (Invitrogen) followed by reverse transcription using the PrimeScript RT reagent Kit (Promega, Madison, WI, USA) according to the manufacturer’s protocol. Real-time PCR was performed using LightCycler480 Real-time PCR system (Roche, Basel, Switzerland). The expression of MiR-371b-5p was determined using the Bulge-Loop™ miRNA kit (RuiBo, Guangzhou, China) with U6 as an endogenous reference. The primers were purchased from Gene Copoeia Co (Guangzhou, China) (Table S2).

### Western blotting assays

Cells were lysed in RIPA buffer (Invitrogen). After clarification by centrifugation, the supernatant proteins were resolved by polyacrylamide-SDS gel electrophoresis and then transferred to polyvinylidene difluoride membrane (Roche) using an electrophoresis system (Bio-Rad, Hercules, CA, USA). The membranes were incubated with primary antibodies overnight and followed by secondary antibodies [28]. The antibodies used were all from Abcam and included anti-GAPDH (1:1000, ab8245), anti-Smad2 (1:1000, ab40855), anti-LEF1 (1:1500, ab137872), anti-caspase-3 (ab32351), anti-cleaved-caspase-3 (ab32042), anti-Bax (1:1000, ab32503), and anti-Bcl-2 antibodies (1:1000, ab32124), and anti-rabbit or anti-mouse IgG (1:5000).

### Lentiviral infection

Vectors expressing miR-371b-5p, miR-371b-5p antisense, miR-371b-5p mimics, mutated miR-371b-5p, Smad2, LINC00183, mutated LINC00183, LINC00183 knocking-down, LINC00183 binding-mutation, β-catenin, LEF1, and LEF1 knocking-down were purchased from Kangcheng Biotechnology (Guangzhou, China). Lentivirus particles were encapsulated with pMDLG/pRRE, pRSV/pREV, pCMV/pVSVG and pLVX-puro vectors. Forty-eight h after transfection using Lipofectamine 2000 reagent (Invitrogen), lentiviruses produced by 293FT cells were collected and filtered. Lentiviruses were introduced into T-LBL cells at the multiplicity of infection of 10 in the presence of 8 mg/mL polybrene (Genecopoeia).

### RNA immunoprecipitation assays

RNA immunoprecipitation assays (RIP) were performed using EZ-Magna RIP RNA-binding protein immunoprecipitation kit (Merck Millipore, MA, USA) according to the manufacturer’s instructions. The cellular lysates were incubated with anti-Argonaute2 (Ago2) antibody (Millipore). Protein A/G magnetic beads combining mouse IgG (Millipore) was used as a negative control. The immunoprecipitated RNA was extracted using phenol: chloroform: isoamyl alcohol (125:24:1) and then analyzed by real-time PCR.

### Chromatin immunoprecipitation (ChIP) assays

The nuclear fraction was extracted using the Nuclear Extraction Kit (Active Motif, CA, USA) and then immunoprecipitated with the ChIP assay kit (Abcam) according to the manufacturer’s instructions. Mouse anti-LEF1 antibody (1:200, ab137872, Abcam) and mouse IgG (Millipore) served as a negative control. Target fragments of promoters containing TCF/LEF1 response element (TRE) were then detected with agarose gel electrophoresis.

### Luciferase reporter assays

The pGL3-Basic vector (Promega) inserted with 3’UTR sequences of *Smad2* and *LEF1*, and other vectors loaded with miR-control, miR-371b-5p-mut, miR-371b-5p-mimic, or anti-miR-371b-5p expression elements were transfected into T-LBL cells. The wildtype or mutated sequence (of which the TCF4/LEF1-binding site was mutated) of LINC00183 promoter was loaded into pGL3-Basic vector followed by transfection into LEF1-overexpression and control T-LBL cells with Renilla luciferase reporter (pRL-TK) as control. pmirGLO vector (Promega) loading LINC00183-wt or LINC00183-mut sequence was transfected into cells. The Renilla and firefly bioluminescence were measured 48 h later using Dual-Luciferase Reporter Kit (Promega).

### Xenograft assays

Animal experiments were conducted with the approval of the Animal Care and Use Committee of SYSUCC and carried out strictly in compliance with the guidelines concerning the handling of experimental animals. In total, 5 × 10^6^ SUP-T1 cells were inoculated on the right flanks of athymic BALB/c nude mice subcutaneously and the volume of the transplanted tumor was measured every 4 days. Thirty-two days post implantation, mice were sacrificed with the xenografts stripped, weighed and photographed.

### Statistical analysis

The expression of lncRNAs in T-LBL patients was examined using *t* test and lncRNAs with a ≥ 2-fold change and p value < 0.05 were considered differentially expressed. Statistical analysis was performed using SPSS software version 17.0 (SPSS Inc., Chicago, IL, USA). Progression-free survival (PFS) was calculated from the date of pathological confirmation to the date of tumor progression or death. Overall survival (OS) was determined from the date of pathological confirmation to the date of death of any cause. The date of the final follow-up visit served as the benchmark for evaluating survival. Survival curves were determined using the Kaplan-Meier method and compared using the log-rank tests. The correlation between two variables was assessed using Pearson’s correlation analysis. *P* value < 0.05 was considered to be statistically significant.

## Results

### LINC00183 is oncogenic and associated with worse clinical outcomes of T-LBL

**Fig. 1:**
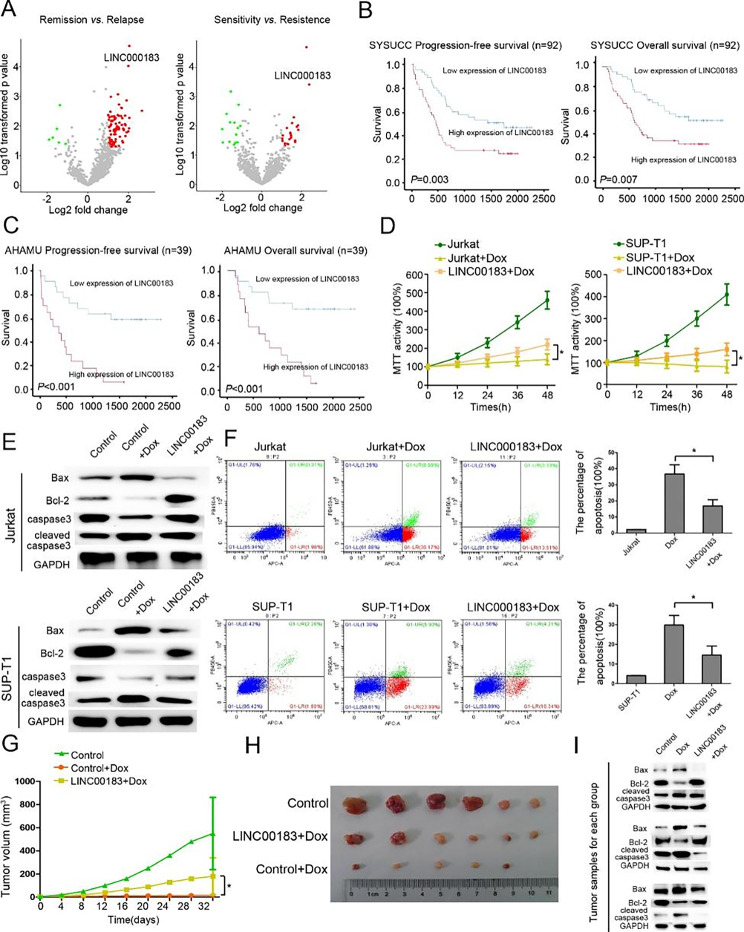
LINC00183 promotes T-LBL progression and chemoresistance *in vivo* and *in vitro. ***A** The differential expression of lncRNAs in 3 remission and relapse tissues (left), and in 3 chemosensitivity and chemoresistance tissues (right). **B** Kaplan-Meier curves of progression-free survival (PFS) and overall survival (OS) stratified by LINC00183 expression in T-LBL patients from the SYSUCC dataset. **C** Kaplan-Meier curves of PFS and OS stratified by LINC00183 expression in T-LBL patients from the AHAMU dataset. **D** MTT assays using T-LBL and LINC00183-overexpression T-LBL cells under the treatment of Dox (100ng/ml). **E** The protein expression of Bax, Bcl-2, caspase-3, and cleaved-caspase-3 in T-LBL and LINC00183-overexpression T-LBL cells treated with Dox (100ng/ml). **F** Flow cytometric analysis of T-LBL cells and LINC00183-overexpression T-LBL under the treatment of Dox (100ng/ml). **G** The tumor growth curve of mouse xenografts. The xenografts were formed SUP-T1 or SUP-T1-LINC00183 cells treated with Dox (1 mg/kg/time, 3 times/week). **H** The tumor size of mouse xenografts. **I** The protein expression of Bax, Bcl-2, and cleaved-caspase-3 in xenograft tissues. 3 xenografts tissues for each group. Dox, doxorubicin. LINC00183, ectopic LINC00183 expression in T-LBL cells. SUP-T1-LINC00183, SUP-T1 cells transfected with LINC00183. *, *P* < 0.05

RNA-sequencing identified 90 differentially expressed lncRNAs between remission T-LBL tissues (from patients who achieved CR after induction chemotherapy and did not relapse within 3 years) and relapse T-LBL tissues (from patients who achieved CR after induction chemotherapy but relapsed within 6 months), and 36 differentially expressed lncRNAs between drug sensitivity T-LBL tissues (from patients who achieved CR after two cycles of induction chemotherapy) and drug-resistant T-LBL tissues (from patients who failed to achieve CR after induction chemotherapy). LINC00183 was the top upregulated lncRNA in T-LBL tissues from patients with relapse or who were chemoresistant (Fig. 1A). Furthermore, the expression of LINC00183 was significantly upregulated in T-LBL patients who relapsed *versus* those who did not and patients who died *versus* those who survived in both the SYSUCC dataset and the AHAMU dataset (Fig. S1). The clinicopathologic characteristics of 39 T-LBL patients from AHAMU dataset and 92 T-LBL patients from the SYSUCC dataset are summarized in Table S1. By using the median expression of LINC00183 as a cutoff for SYSUCC dataset, Kaplan-Meier analysis revealed that T-LBL patients with high LINC00183 expression had significantly worse PFS (HR 2.21, 95%CI 1.30–3.76, p = 0.003) and OS (HR 2.17, 95%CI 1.23–3.82, p = 0.007) than those with low LINC00183 expression (Fig. 1B). In the AHAMU cohort, patients with high LINC00183 expression had significantly poorer PFS (HR 4.61 95%CI 1.98–10.75, p < 0.001) and OS (HR 5.03, 95%CI 2.04–12.41, p < 0.001) than those with low LINC00183 expression (Fig. 1C).

We further investigated the effect of LINC00183 on chemoresistance of T-LBL cells. We found that the expression of LINC00183 in Jurkat and SUP-T1 cells was higher compared to in normal T cells (Fig. S2). We infected Jurkat and SUP-T1 cells with lentiviruses overexpressing or knocking down LINC00183 (Fig. S2). The appropriate dosage of doxorubicin (Dox) was used to induce cell apoptosis (Fig. S3). MTT assays showed that LINC00183 overexpression significantly reduced the viabilities of Jurkat and SUP-T1 cells (Fig. 1D). Western blotting assays further showed that LINC00183 overexpression downregulated Bax and cleaved-caspase-3 expression while upregulating Bcl-2 and caspase-3 expression in Jurkat cells compared to controls under the treatment of Dox (Fig. 1E). Flow cytometric analysis further demonstrated a dramatic reduction in the proportion of apoptotic cells upon doxorubicin induction of LINC00183 expression (Fig. 1F).

Mouse xenograft assays showed that the tumor volume of xenografts bearing SUP-T1 cells overexpressing LINC00183 was considerably greater than that of control xenografts (Fig. 1G, H). Immunoblotting assays and immumohistochemical staining assays showed a noticeable rise in Bcl-2 and Ki67 expression and a reduction in Bax and cleaved-caspase-3 expression upon induction of LINC00183 with doxorubicin in mouse xenografts bearing SUP-T1 cells (Fig. 1I, Fig. S4).

### LINC00183 targets miR-371b-5p in T-LBL

**Fig. 2:**
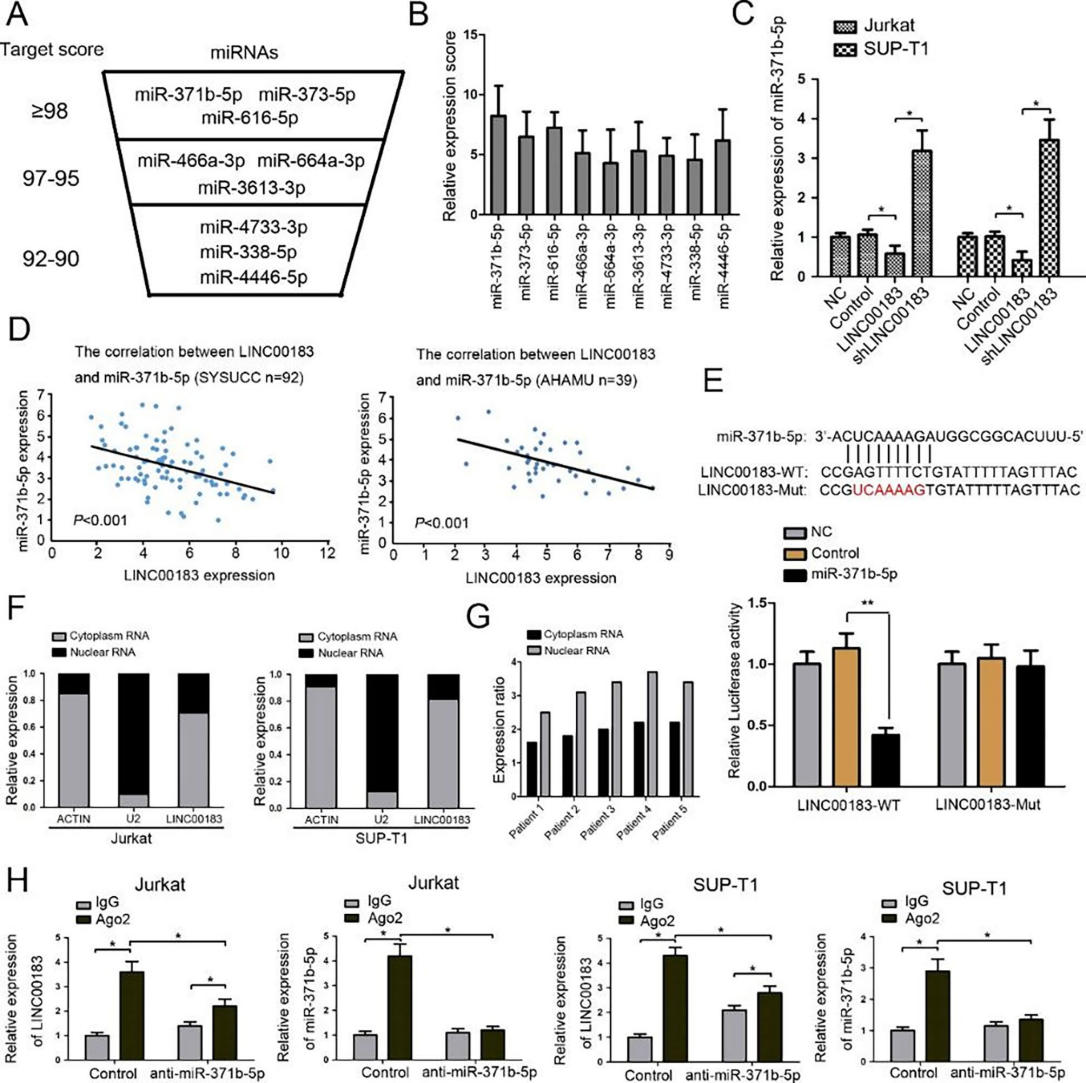
MiR-371b-5p is regulated by LINC00183. **A** LINC00183 capable of regulating miRNAs was predicted by bioinformatic tool miRDB. **B** The relative expression score (miRNAs level/LINC00183 level) was calculated in 9 predicted miRNAs. **C** The expression of miR-371b-5p in T-LBL cells transfected by LINC00183 and shLINC00183. **D** Correlation between LINC00183 gene expression and miR-371b-5p level in the SYSUCC and AHAMU dataset. **E** The predicted binding sites between miR-371b-5p and LINC00183. The mutated site (LINC00183-mut) was used for luciferase reporter assays. The relative luciferase activities were detected in 293T cells transfected by LINC00183-WT and LINC00183-mut. **F** LINC00183 is abundant in the cytoplasm of Jurkat and SUP-T1 cells. U2 and actin were used as positive control. **G** Cytoplasmic enrichment of LINC00183 in T-LBL tissues. **H** RNA-IP was used to identify inhibition of miR-371b-5p by LINC00183. The expression levels of LINC00183 and miR-371b-5p were detected using quantitative RT-PCR. LINC00183, ectopic LINC00183 expression in T-LBL cells. shLINC00183, T-LBL cells were transfected by shRNA targeting LINC00183. Dox, doxorubicin. *, *P* < 0.05

We identified potential targeting miRNAs of LINC00183 by using the bioinformatic tool miRDB [29]. We selected the top 9 predicted miRNAs with miRDB target score ≥ 92 (Fig. 2A). We used the relative expression score (the ratio of LINC00183 to miRNA expression) to evaluate which miRNA was the most possible target of LINC00183. We found that miR-371b-5p was most significantly associated with LINC00183 among the 9 miRNAs (Fig. 2B). Overexpression of LINC00183 dramatically decreased whereas knockdown of LINC00183 by shLINC00183 significantly increased miR-371b-5p levels in T-LBL cells (Fig. 2C). Pearson correlation analysis demonstrated a significant negative correlation between miR-371b-5p and LINC00183 in the SYSUCC and AHAMU datasets (Fig. 2D). As further revealed by the luciferase reporter assays, co-transfection of LINC00183-wt and miR-371b-5p led to significantly reduced bioluminescence whereas co-transfection of LINC00183-mut and miR-371b-5p had no obvious impact on luciferase activity compared to their specific controls (Fig. 2E). In both T-LBL cells and tissues, subcellular fractionation analysis showed that LINC00183 was primarily present in the cytoplasm (Fig. 2F, G). RIP assays were performed to further explore the mechanisms under which LINC00183 regulated miR-371b-5p. The amount of LINC0183 and miR-371b-5p immunoprecipitated with Ago2 was greater in the control group than that precipitated with isotype IgG control. LINC00183 and miR-371b-5p immunoprecipitated with Ago2 were considerably less abundant in the anti-miR-371b-5p group compared to the control group (Fig. 2H). Particularly, with anti-miR-371b-5p treatment, the amount of LINC0183 was higher than the IgG group, suggesting that miR-371b-5p might have inhibitory feedback on LINC00183 expression.

### MiR-371b-5p mediates LINC00183-related chemoresistance

**Fig. 3:**
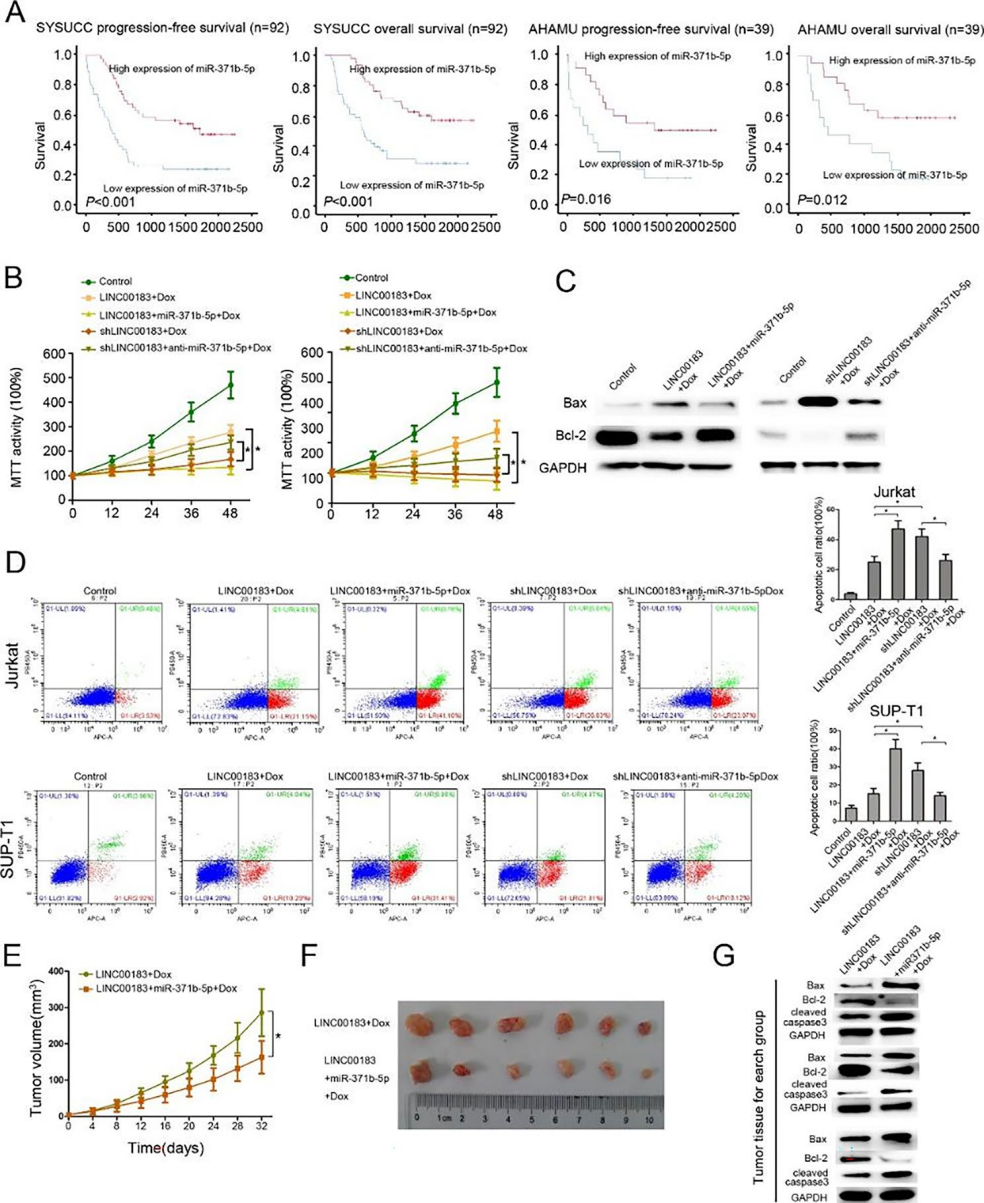
miR-371b-5p suppresses T-LBL progression and chemoresistance *in vivo* and *in vitro. ***A** Kaplan-Meier curves of progression-free survival (RFS) and OS stratified by miR-371b-5p expression in patients from the SYSUCC and AHAMU datasets. **B** MTT assay using T-LBL cells transfected by LINC00183, LINC00183 + miR-371b-5p, shLINC00183, shLINC00183 + anti-miR-371b-5p treated with Dox (100ng/ml). **C** The protein levels of Bax, Bcl-2 in SUP-T1 cells transfected by LINC00183, and LINC00183 + miR-371b-5p treated with Dox (100ng/ml). **D** Flow cytometric analysis of T-LBL cells transfected by LINC00183, shLINC00183, LINC00183 + miR-371b-5p, and shLINC00183 + anti-miR-371b-5p treated with Dox (100ng/ml). **E** The tumor growth curve of mouse xenografts. The xenografts were formed SUP-T1-LINC00183 or SUP-T1-LINC00183 + miR-371b-5p cells treated with Dox (1 mg/kg/time, 3 times/week). **F** The tumor size of mouse xenografts. **G** The protein expression of Bax, Bcl-2, and cleaved-caspase-3 in xenograft tissues. 3 xenografts tissues for each group. LINC00183, ectopic LINC00183 expression in T-LBL cells. shLINC00183, T-LBL cells were transfected by shRNA targeting LINC00183. LINC00183 + miR-371b-5p, T-LBL cells were transfected with LINC00183 and miR-371b-5p. shLINC00183 + anti-miR-371b-5p, T-LBL cells were transfected with shLINC00183 and anti-miR-371b-5p. Dox, doxorubicin. *, *P* < 0.05

We next examined the correlation between miR-371b-5p expression and survival in the SYSUCC and the AHAMU datasets. RT-PCR assays showed significant miR-371b-5p downregulation in T-LBL tissues of patients who relapsed or died compared to T-LBL tissues of patients who did not relapse or who survived in both datasets (n = 39) (*P* < 0.05) (Fig. S5). In addition, using the median expression of miR-371b-5p as a cutoff, patients with low miR-371b-5p expression had significantly worse OS and PFS than those with high miR-371b-5p expression in the SYSUCC and AHAMU datasets (Fig. 3A).

We further explored the function of miR-371b-5p on apoptosis-resistance of LINC00183 in vitro and *in vivo.* We constructed Jurkat and SUP-T1 stable cells overexpressing miR-371b-5p or with miR-371b-5p knockdown *via* lentivirus infection (Fig. S6). MTT assays, Western blotting assays, and flow cytometry revealed that miR-371b-5p overexpression dramatically increased apoptosis of cells overexpressing LINC00183. In addition, LINC00183 knockdown with shRNAs remarkedly increased the apoptosis of T-LBL cells, which was abated by miR-371b-5p inhibitors (Fig. 3B-D).

Xenograft assays showed that miR-371b-5p overexpression reduced the growth of xenografts bearing SUP-T1 cells overexpressing LINC00183 and the tumor volume on day 32 was significantly smaller than that of xenografts bearing SUP-T1 cells overexpressing LINC00183 only (Fig. 3E, F). Western blotting and immumohistochemical staining assays showed that miR-371b-5p decreased Bax, cleaved-caspase-3 levels and increased Bcl-2 and Ki67 levels in xenografts bearing SUP-T1 cells overexpressing LINC00183 (Fig. 3G, Fig. S7).

### MiR-371b-5p targets Smad2/LEF1 in T-LBL

**Fig. 4:**
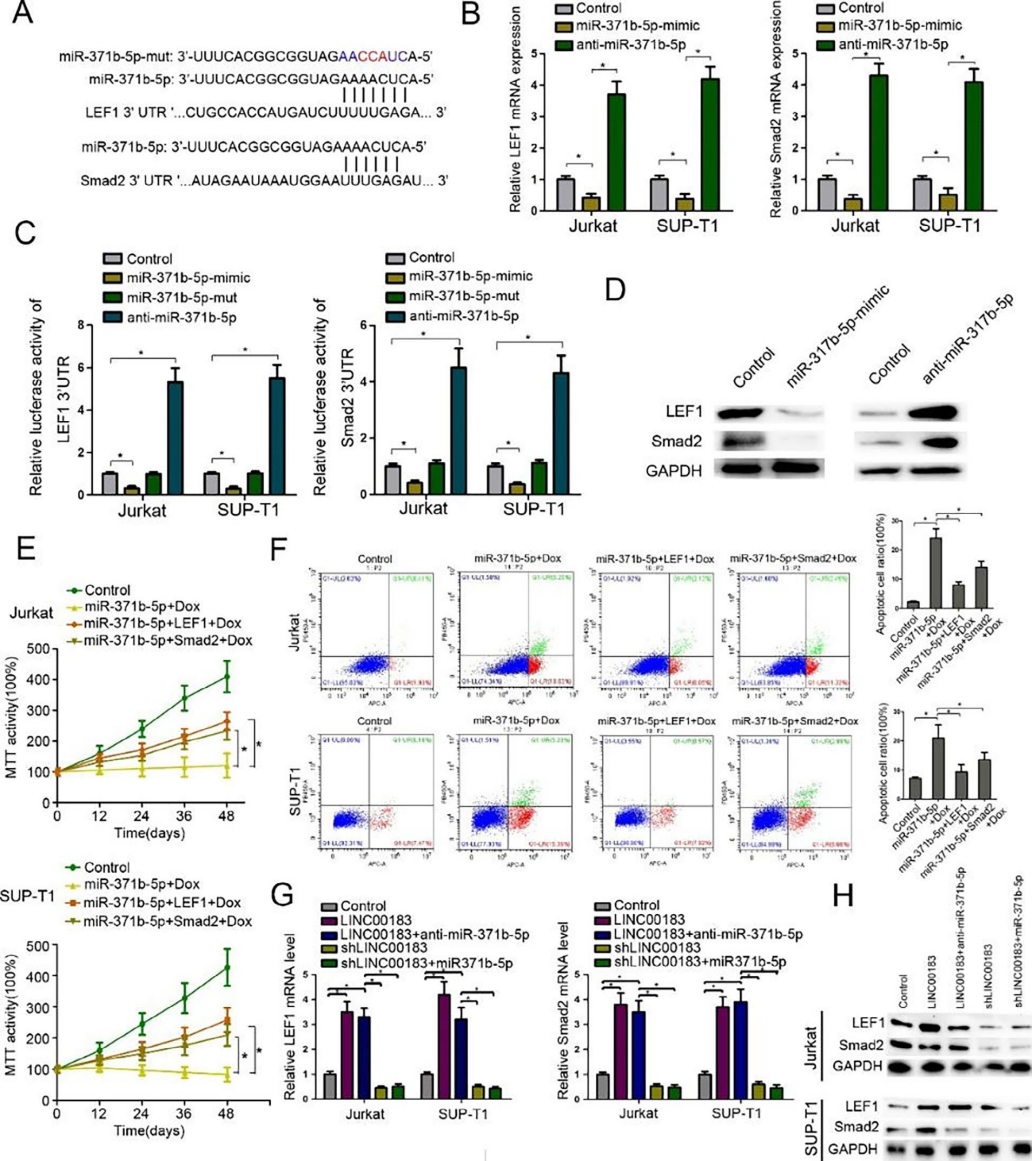
MiR-371b-5p targets Smad2/LEF1 in T-LBL cells. **A** The predicted target sequence of miR-371b-5p in the 3’UTR of *Smad2* (Smad2-3’UTR) and *LEF1* (LEF1-3’UTR) and mutant containing three altered nucleotides in the seed sequence of miR-371b-5p (miR-371b-5p-mut). **B** Smad2/LEF1 mRNA levels in T-LBL cells after transfection with miR-371b-5p mimics and anti-miR-1258. **C** Luciferase assays of pGL3-Smad2-3’UTR and pGL3-LEF1-3’UTR in the presence of miR-371b-5p mimics, miR-371b-5p -mutant, and anti-miR-371b-5p. **D** The protein levels of Smad2 and LEF1 after transfection of T-LBL cells with miR-1258 mimics and anti-miR-1258. **E** MTT assays using T-LBL cells transfected by miR-371b-5p, miR-371b-5p + LEF1, miR-371b-5p + Smad2 treated with Dox (100ng/ml). **F** Flow cytometric analysis of T-LBL cells transfected with miR-371b-5p, miR-371b-5p + LEF1, miR-371b-5p + Smad2 treated with Dox (100ng/ml). **G** Smad2/LEF1 mRNA levels after transfection with LINC00183, LINC00183 + anti-miR-371b-5p, shLINC00183 and shLINC00183 + miR-371b-5p. **H** The protein levels of Smad2 and LEF1 after transfection of T-LBL cells with LINC00183, LINC00183 + anti-miR-371b-5p, shLINC00183 and shLINC00183 + miR-371b-5p. LINC00183, ectopic LINC00183 expression in T-LBL cells. shLINC00183, T-LBL cells were transfected by shRNA targeting LINC00183. LINC00183 + anti-miR-371b-5p, T-LBL cells were transfected with LINC00183 and anti-miR-371b-5p. shLINC00183 + miR-371b-5p, T-LBL cells were transfected with shLINC00183 and miR-371b-5p. Dox, doxorubicin. *, *P* < 0.05

Online miRNA target prediction database (Targetscan) was used to identify potential miR-371b-5p target genes [30], we identified both probable miR-371b-5p binding sites in *Smad2* and *LEF1*, two positive regulators of tumor progression (Fig. 4A). RT-PCR revealed that miR-371b-5p-mimics dramatically suppressed while anti-miR-371b-5p significantly increased the mRNA levels of *Smad2* and *LEF1* in T-LBL cells (Fig. 4B). MiR-371b-5p-mimics dramatically decreased the luciferase activities of Smad2-3’UTR (luc-Smad2-3’UTR) and LEF1-3’UTR (luc-LEF1-3’UTR), which were reversed by miR-371b-5p-mut and remarkably increased by antisense miR-371b-5p (Fig. 4C). Additionally, Western blotting assays demonstrated that miR-371b-5p mimics notably reduced Smad2 and LEF1 levels which was abolished by anti-miR-371b-5p (Fig. 4D). MTT assays showed that Smad2 or LEF1 significantly increased the viabilities of T-LBL cells compared with cells treated with miR-371b-5p mimics alone (Fig. 4E) and miR-371b-5p mimics noticeably increased the proportion of apoptotic T-LBL cells *versus* controls, which was significantly abated by addition of Smad2 or LEF1 (Fig. 4F). We further examined the effect of LINC00183 on the expression of Smad2 and LEF1. LINC00183 overexpression significantly upregulated while shLINC00183 remarkedly repressed both the mRNA and protein levels of Smad2 and LEF1 in T-LBL cells (Fig. 4G). Meanwhile, miR-371b-5p mimics or anti-miR-371b-5p could reverse LINC00183-mediated changes in Smad2 and LEF1 in T-LBL cells (Fig. 4H).

### LINC00183 is a connector between the TGF-β/Smad and the Wnt/β-catenin signaling pathways

**Fig. 5:**
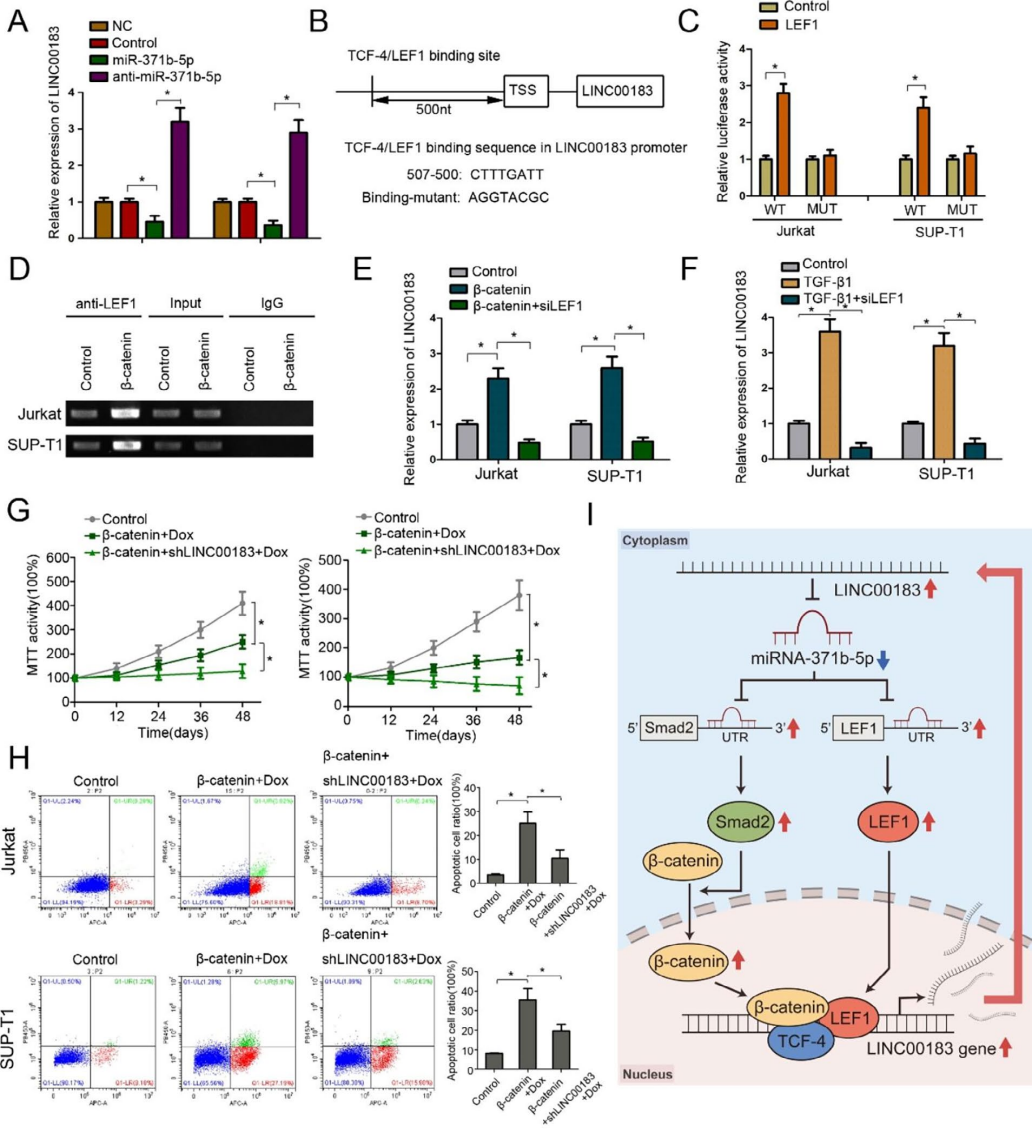
LINC01278 is regulated by the Wnt/β-catenin pathway. **A** The expression of LINC00183 in T-LBL cells transfected with miR-371b-5p and anti-miR-371b-5p. **B** Identification of TCF-4/LEF-1 binding site in the LINC00183’s promoter sequence. The mutant sequence is designed for luciferase reporter assay. **C** The relative luciferase activity was detected in 293T cells co-transfected with Binding-WT/Binding-mut and LEF1. **D** ChIP assay was used to detect the binding of LEF1 to the TRE (TCF responsive element: TTCAAAG) region in the LINC00183 promoter. **E** The relative expression of LINC00183 in T-LBL cells transfected with β-catenin and siLEF1-4. **F** The relative expression of LINC00183 in T-LBL cells treated with TGF-β1 (0.5 ng/ml) and siLEF1. **G** MTT assays of T-LBL cells transfected with β-catenin and shLINC00183 treated with Dox (100ng/ml). **H** Flow cytometric analysis of apoptotic T-LBL cells transfected with β-catenin and shLINC00183 treated with Dox (100ng/ml). **I** Schematic illustration of the β-catenin-LINC00183-miR-371b-5p-Smad2/LEF1 axis. The up-regulation of LINC00183 reduce the expression of miR-371b, thereby up-regulate the expression of Smad2 and LEF1 proteins. Smad2 can promote the entry of β-catenin protein into the nucleus, and facilitate the formation of transcription complex between β-catenin, TCF4 and LEF1, and positively feedback the level of transcription LINC0083. The blue arrows indicate downregulated; the red arrows indicate upregulated. LINC00183, ectopic LINC00183 expression in T-LBL cells. shLINC00183, T-LBL cells were transfected by shRNA targeting LINC00183. β-catenin, T-LBL cells were transfected with β-catenin. β-catenin + shLINC00183, T-LBL cells were transfected with shLINC00183 and β-catenin. siLEF1, T-LBL cells were transfected by siRNA targeting LEF1. Dox, doxorubicin. *, *P* < 0.05

The results of RIP assays prompted us to speculate that miR-371b-5p could have an inhibitory feedback impact on the expression of LINC00183 (Fig. 2H). RT-PCR assays showed that forced expression of miR-371b-5p dramatically repressed the expression of LINC00183 (Fig. 5A). Analysis of the promoter sequence of LINC00183 using the online tool PROMO revealed a potential TCF-4/LEF1 binding site (Fig. 5B) [31]. Additionally, luciferase reporter assays demonstrated that co-transfection of LINC00183-wt and LEF1 dramatically elevated the luciferase activities compared with the control group in T-LBL cells, whereas co-transfection of LEF1 and LINC00183-mut (of which the TCF-4/LEF1 binding sequence on the promoter was mutated) had no significant impact on luciferase activities compared to their controls (Fig. 5C). ChIP assays further showed that LEF1 could bind to the promoter of LINC00183. Moreover, the binding of LEF1 to LINC00183 promoter was increased with β-catenin overexpression (Fig. 5D), leading to the upregulation of LINC00183 expression in T-LBL cells, which was aborted by co-transfection with siLEF1 (Fig. 5E). Additionally, TGF-β1 upregulated the expression of LINC00183, which, however, was abated by co-transfection with siLEF1 (Fig. 5F). MTT assays and flow cytometry showed that forced expression of β-catenin reduced the survival and promoted the apoptotic death of T-LBL cells, which was accentuated by shLINC00183 (Fig. 5G, H). Treatment with small molecular inhibitor targeting Wnt/β-catenin signaling reduced the expression of LINC00183 (Fig. S8) and attenuated the growth inhibitory effects of LINC00183 (Fig. S9). These findings demonstrated that LINC00183 might serve as a connector between the TGF-β/Smad and the Wnt/β-catenin signaling pathways in T-LBL.

## Discussion

In this work, we revealed that upregulation of LINC00183 expression in chemoresistant T-LBL samples, and LINC00183 was associated with poor OS and PFS in adult T-LBL patients. Moreover, in vivo and in vitro assays showed that LINC00183 was a key player in the β-catenin/LEF1-LINC00183-miR-371b-5p-Smad2/LEF1 axis regulating T-LBL progression and chemoresistance. These findings imply that targeting LINC00183 could represent a potential therapeutic approach for preventing T-LBL progression and drug resistance.

Emerging evidence shows that lncRNAs act as internal functional and regulatory constituents in many cancers. Only one T-LBL-related lncRNA study reported that maternally expressed gene 3 (MEG3) regulate T-LBL tumorigenesis and epithelial-mesenchymal transition (EMT) by activating the PI3K/mTOR signaling pathway [21]. However, it did not carry out genome-wide lncRNA screening, which may have missed important lncRNAs. In this study, we carried out RNA sequencing to screen key lncRNAs that impact T-LBL progression and chemoresistance. We found that LINC00183 was the top upregulated lncRNA in both relapse and drug-resistant T-LBL tissues. LINC00183, located on chromosome X:73,948,346–73,952,178, is a novel lncRNA whose function has not yet been reported. We found that LINC00183 was associated with T-LBL prognosis in both the SYSUCC dataset and the AHAMU dataset. The results of i*n vitro* and in vivo experiments revealed that the expression of LINC00183 positively correlated with T-LBL progression and drug resistance.

According to the “ceRNA” hypothesis, lncRNA may control mRNA expression through sponging miRNAs [17]. Here, we searched for potential miRNAs that may mediate the function of LINC00183 using the online prediction tool. We found that miR-371b-5p was a potential target of LINC00183. MiR-371b-5p is reported to regulate cell proliferation, cell cycle progression and tumor metastasis in many types of cancer [32, 33]. We found that miR-371b-5p expression negatively correlated with LINC00183 expression in the SYSUCC and AHAMU datasets. In addition, miR-371b-5p was positively associated with T-LBL prognosis in both cohorts. The results of i*n vivo* and in vitro experiments revealed that miR-371b-5p could reduce LINC00183-induced chemoresistance, suggesting that LINC00183 promoted T-LBL chemoresistance depending on miR-371b-5p expression. We used the online tools to further screen potential targets of miR-371b-5p, identifying both Smad2 and LEF1. According to the luciferase reporter assays, miR-371b-5p binds to the 3’-UTR of Smad2 and LEF1, preventing the expression of these genes. In the classic pattern, TGF-β binding to its receptors activates SMADs and promotes the formation and translocation of Smad complex [34]. Smad complex controls numerous target genes with the assistance of co-factors through binding to the gene promoters [34]. LEF1 is an important downstream mediator of the Wnt/β-catenin signaling pathway [35] and has been linked to carcinogenesis, cancer proliferation, migration, and invasion [35]. MiR-371b-5p targets Smad2/LEF1 specifically, blocks the TGF-β/Smad and Wnt/β-catenin signaling pathway, and inhibits its function.

Previous literature reported that RISC, the complex participating in microRNA or siRNA-mediated gene silencing, is a crucial point in the “ceRNA” pattern with Ago2 as a foundational element of RISC [36]. We then investigated the potential mechanism under which LINC00183 targeted miR-371b-5p through RIP assays. The data showed that the amount of miR-371b-5p and LINC00183 immunoprecipitated with Ago2 increased. The amount of LINC00183 immunoprecipitated with Ago2 dramatically increased following the upregulation of miR-371b-5p, suggesting that LINC00183 could mediate miR-371b-5p by RISC complex.

We also found that miR-371b-5p could reciprocally regulate the level of LINC00183. Using the online tool PROMO, we searched for the potential factor binding sites [31]. According to analysis, transcription factors LEF-1 and TCF-4 were suggested to bind to the promoter of LINC00183. The putative binding site was then validated by luciferase reporter assays and ChIP assays. Then, quantitative RT-PCR analysis further revealed that upregulation of either β-catenin or TGF-β1 could increase LINC00183 levels. Furthermore, downregulation of LINC00183 could suppress chemoresistance induced by β-catenin. Previous studies have reported that β-catenin could be maintained by the Smad2/3 complex in the cytoplasm and promoted to translocate to the nucleus. Many studies have also shown that the Wnt signaling pathway plays an important role in T-LBL. Therefore, the LINC00183-miR-371b-5p-Smad2/LEF1 axis could in turn activate the Wnt/β-catenin signaling pathway, and then promote the expression of LINC00183, creating a positive feedback loop to amplify its impact in T-LBL.

## Conclusions

Our findings indicated that the LINC00183-miR-371b-5p-Smad2/LEF1 axis promotes T-LBL drug-resistance. This axis increases the stability of Wnt/β-catenin signaling and forms a positive feedback loop, which further promotes T-LBL drug-resistance. LINC00183 as a crucial connector between Wnt signaling and TGF-β/Smad signaling may be developed to be a new therapeutic target to prevent drug-resistance in T-LBL.

## Electronic supplementary material

Below is the link to the electronic supplementary material.

Supplementary Material 1

Supplementary Material 2

Supplementary Material 3

Supplementary Material 4

Supplementary Material 5

Supplementary Material 6

Supplementary Material 7

Supplementary Material 8

Supplementary Material 9

Supplementary Material 10

Supplementary Material 11

Supplementary Material 12

## Data Availability

The key raw data have been uploaded onto the Research Data Deposit public platform (RDD), with the approval RDD number of RDDB2022480441.

## Abbreviations

T-LBL: T-cell lymphoblastic lymphoma
lncRNAs: long non-coding RNAs
DFS: disease-free survival
CR: complete remission
ncRNAs: Non-coding RNAs
ceRNA: competitive endogenous RNA
MREs: miRNA-response elements
LEF1: Lymphoid enhancer-binding factor 1
FFPE: Formalin-fixed, paraffin-embedded
SYSUCC: Sun Yat-sen University Cancer Center
AHAMU: Affiliated Hospital of Anhui Medical University
PET-CT: Positron emission tomography computed tomography
RIP: RNA immunoprecipitation assays
ChIP: Chromatin immunoprecipitation
TRE: TCF/LEF1 response element
PFS: Progression-free survival
OS: Overall survival
EMT: epithelial-mesenchymal transition
Dox: doxorubicin
