# Supplementary material for: The β-catenin-LINC00183-miR-371b-5p-Smad2/LEF1 axis promotes adult T-cell lymphoblastic lymphoma progression and chemoresistance
Source: J Exp Clin Cancer Res. 2023 Apr 28;42:105. doi: 10.1186/s13046-023-02670-9 (PMC10141948; doi:10.1186/s13046-023-02670-9)
Supplement: Supplementary file 1 — Supplementary Material 1 [file 13046_2023_2670_MOESM1_ESM.docx]

Table S1. The clinicopathologic characteristics of T-cell lymphoblastic lymphoma (T-LBL) patients stratified by expression of LINC00183 and miR-371b-5p in SYSUCC dataset and AHAMU dataset.

| Variable | Total cases | SYSUCC dataset | | | | Total cases |  | |  | | AHAMU dataset | |
| --- | --- | --- | --- | --- | --- | --- | --- | --- | --- | --- | --- | --- |
|  |  | High expression of LINC00183 | Low expression of LINC00183 | High expression of miR-371b-5p | Low expression of miR-371b-5p |  | High expression of LINC00183 | Low expression of LINC00183 | | High expression of miR-371b-5p | | Low expression of miR-371b-5p |
| Age (years) |  |  |  |  |  |  |  |  | |  | |  |
| ＞45 | 16 | 2 | 14 | 8 | 8 | 10 | 6 | 4 | | 5 | | 5 |
| ≤45 | 76 | 45 | 31 | 38 | 38 | 29 | 11 | 18 | | 17 | | 12 |
| Gender |  |  |  |  |  |  |  |  | |  | |  |
| Male | 68 | 36 | 32 | 34 | 34 | 28 | 13 | 15 | | 15 | | 13 |
| Female | 24 | 11 | 13 | 12 | 12 | 11 | 4 | 7 | | 7 | | 4 |
| ECOG-PS |  |  |  |  |  |  |  |  | |  | |  |
| <2 | 80 | 41 | 39 | 41 | 39 | 32 | 12 | 20 | | 19 | | 13 |
| ≥2 | 12 | 6 | 6 | 5 | 7 | 7 | 5 | 2 | | 3 | | 4 |
| Effusion, pleural and/or pericardia |  |  |  |  |  |  |  |  | |  | |  |
| Yes | 63 | 33 | 30 | 32 | 31 | 25 | 11 | 14 | | 14 | | 11 |
| No | 29 | 14 | 15 | 14 | 15 | 14 | 6 | 8 | | 8 | | 6 |
| Mediastinal involvement |  |  |  |  |  |  |  |  | |  | |  |
| Yes | 82 | 41 | 41 | 44 | 38 | 36 | 17 | 19 | | 20 | | 16 |
| No | 10 | 6 | 4 | 2 | 8 | 3 | 0 | 3 | | 2 | | 1 |
| Bone marrow involvement |  |  |  |  |  |  |  |  | |  | |  |
| Yes | 28 | 15 | 13 | 11 | 17 | 12 | 5 | 7 | | 7 | | 5 |
| No | 64 | 32 | 32 | 35 | 29 | 27 | 12 | 15 | | 15 | | 12 |
| CNS involvement |  |  |  |  |  |  |  |  | |  | |  |
| Yes | 3 | 3 | 0 | 1 | 2 | 3 | 2 | 1 | | 2 | | 1 |
| No | 89 | 44 | 45 | 45 | 44 | 36 | 15 | 21 | | 20 | | 16 |
| LDH concertation |  |  |  |  |  |  |  |  | |  | |  |
| normal | 30 | 13 | 17 | 17 | 13 | 15 | 6 | 9 | | 11 | | 4 |
| elevated | 62 | 34 | 28 | 29 | 33 | 24 | 11 | 13 | | 11 | | 13 |
| Ann Arbor Stage |  |  |  |  |  |  |  |  | |  | |  |
| ≤2 | 80 | 43 | 37 | 40 | 40 | 32 | 15 | 17 | | 18 | | 14 |
| ＞2 | 12 | 4 | 8 | 6 | 6 | 7 | 2 | 5 | | 4 | | 3 |
| Relapse |  |  |  |  |  |  |  |  | |  | |  |
| Yes | 58 | 35 | 23 | 23 | 35 | 25 | 16 | 9 | | 11 | | 14 |
| No | 34 | 12 | 22 | 23 | 11 | 14 | 1 | 13 | | 11 | | 3 |

T-LBL: T-cell lymphoblastic lymphoma; CNS central nervous, ECOG-PS Eastern Cooperative Oncology Group performance status, LDH lactate dehydrogenase.
