# Supplementary material for: The β-catenin-LINC00183-miR-371b-5p-Smad2/LEF1 axis promotes adult T-cell lymphoblastic lymphoma progression and chemoresistance
Source: J Exp Clin Cancer Res. 2023 Apr 28;42:105. doi: 10.1186/s13046-023-02670-9 (PMC10141948; doi:10.1186/s13046-023-02670-9)
Supplement: Supplementary file 2 — Supplementary Material 2 [file 13046_2023_2670_MOESM2_ESM.docx]

Table S2. The sequence of primers.

| Gene name | Sequence |
| --- | --- |
| Smad-2 | Forward: 5’- TTCAGTTCCGCCTCCAATCG -3’: |
|  | Reverse: 5’- AGCAAGCCACGCTAGGAAAA -3’: |
| LEF1 | Forward: 5’- CCCGTGAAGAGCAGGCTAAA -3’: |
|  | Reverse: 5’- AGGCAGCTGTCATTCTTGGA -3’: |
| LEF1 siRNA | SS sequence 5’- GGUGUUCAGUAGAGCUAAAUA -3’: |
|  | As sequence 5’- UUUAGCUCUACUGAACACCUU -3’: |
| LINC00183 | Forward: 5’- TTGCAAGGCGTCCGAAGTAT -3’: |
|  | Reverse: 5’- AGGCGATCAGCGAGAAAGAA -3’: |
| shLINC00183 | SS sequence 5’- GCGAGACTCTGTCTCTAAA -3’: |
|  | As sequence 5’- TTTAGAGACAGAGTCTCGC -3’: |
