# Supplementary figures and images for: The β-catenin-LINC00183-miR-371b-5p-Smad2/LEF1 axis promotes adult T-cell lymphoblastic lymphoma progression and chemoresistance
Source: J Exp Clin Cancer Res. 2023 Apr 28;42:105. doi: 10.1186/s13046-023-02670-9 (PMC10141948; doi:10.1186/s13046-023-02670-9)

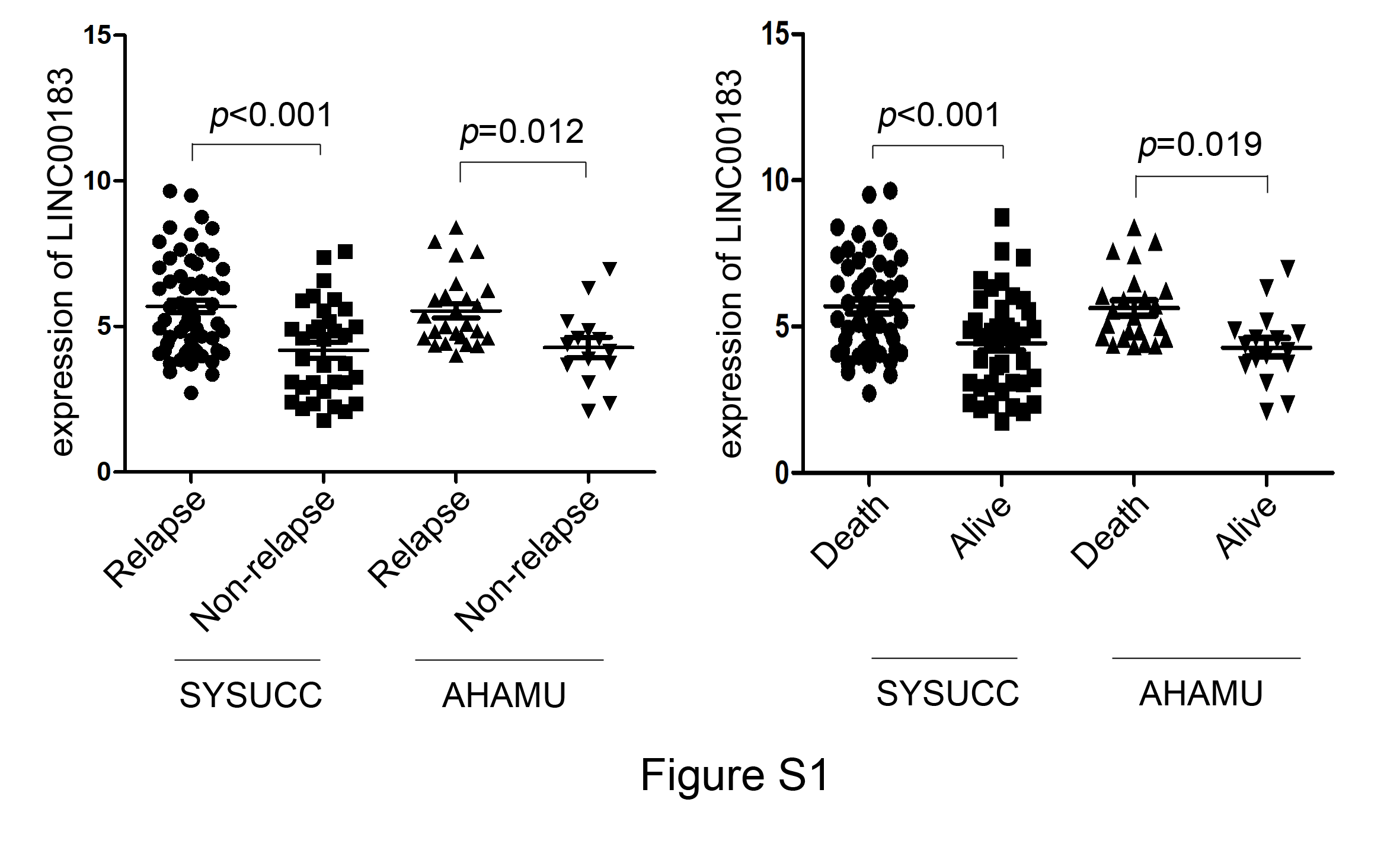

Supplement: Supplementary file 3 — Supplementary Material 3 [file 13046_2023_2670_MOESM3_ESM.tif]

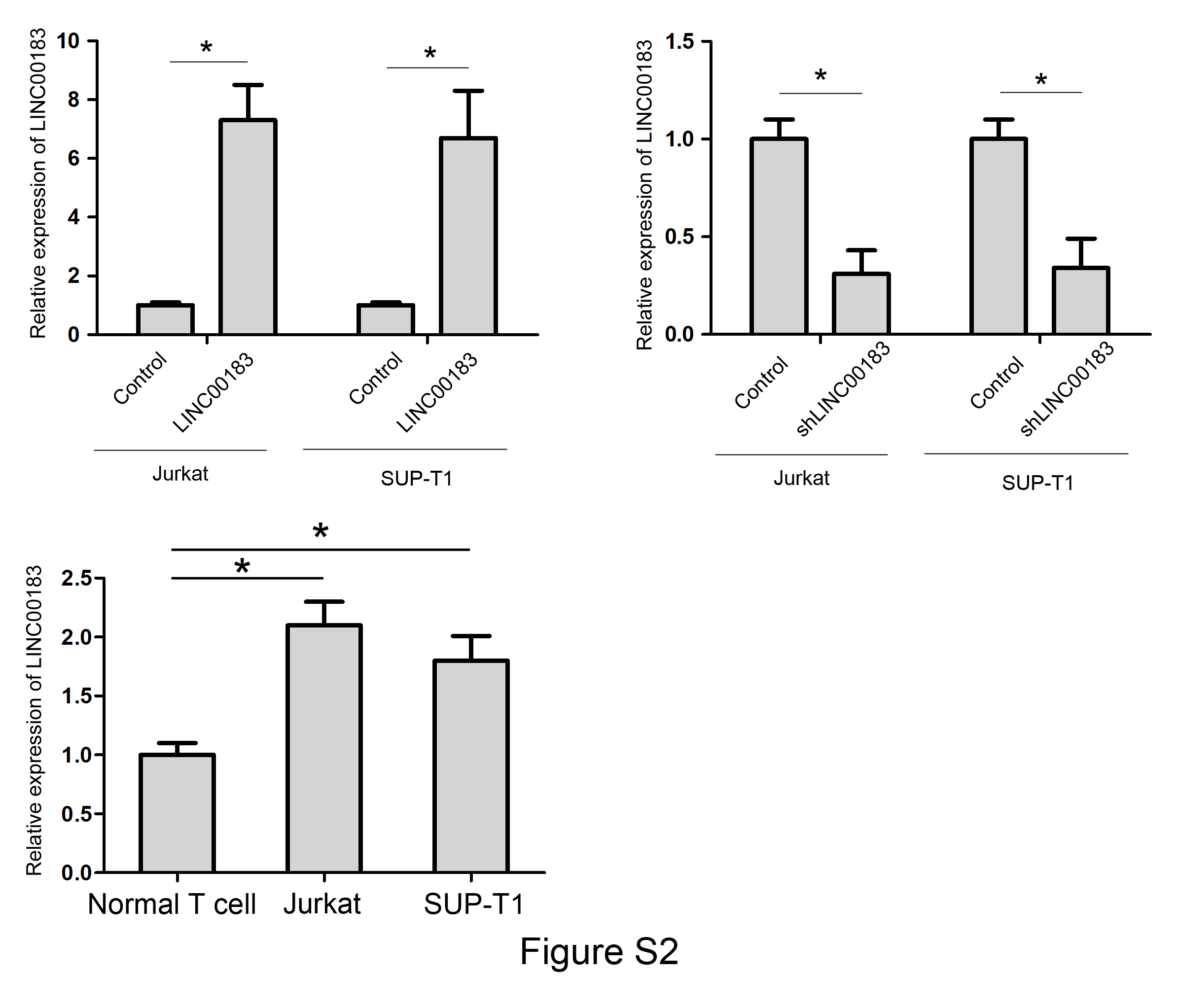

Supplement: Supplementary file 4 — Supplementary Material 4 [file 13046_2023_2670_MOESM4_ESM.tif]

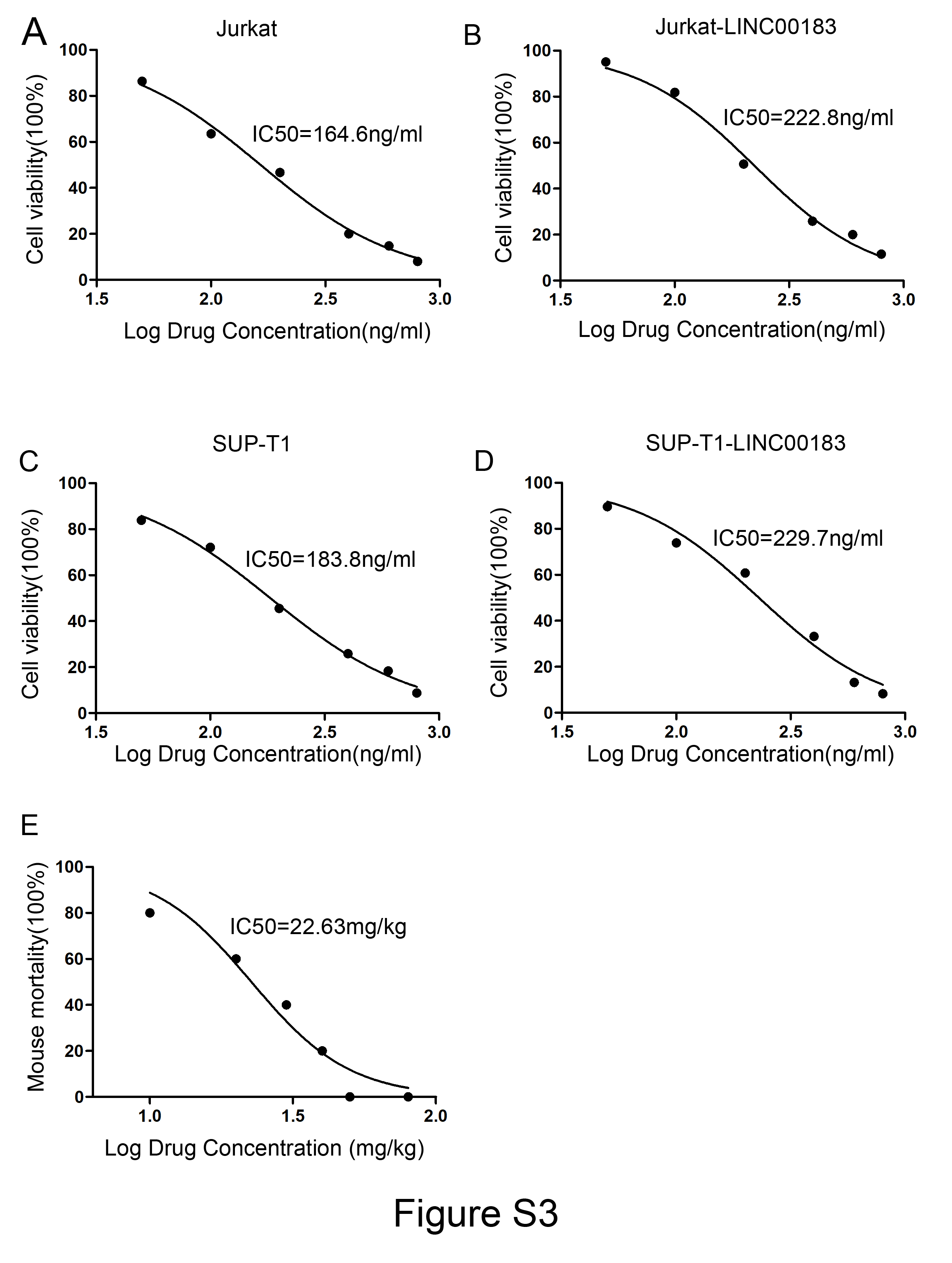

Supplement: Supplementary file 5 — Supplementary Material 5 [file 13046_2023_2670_MOESM5_ESM.tif]

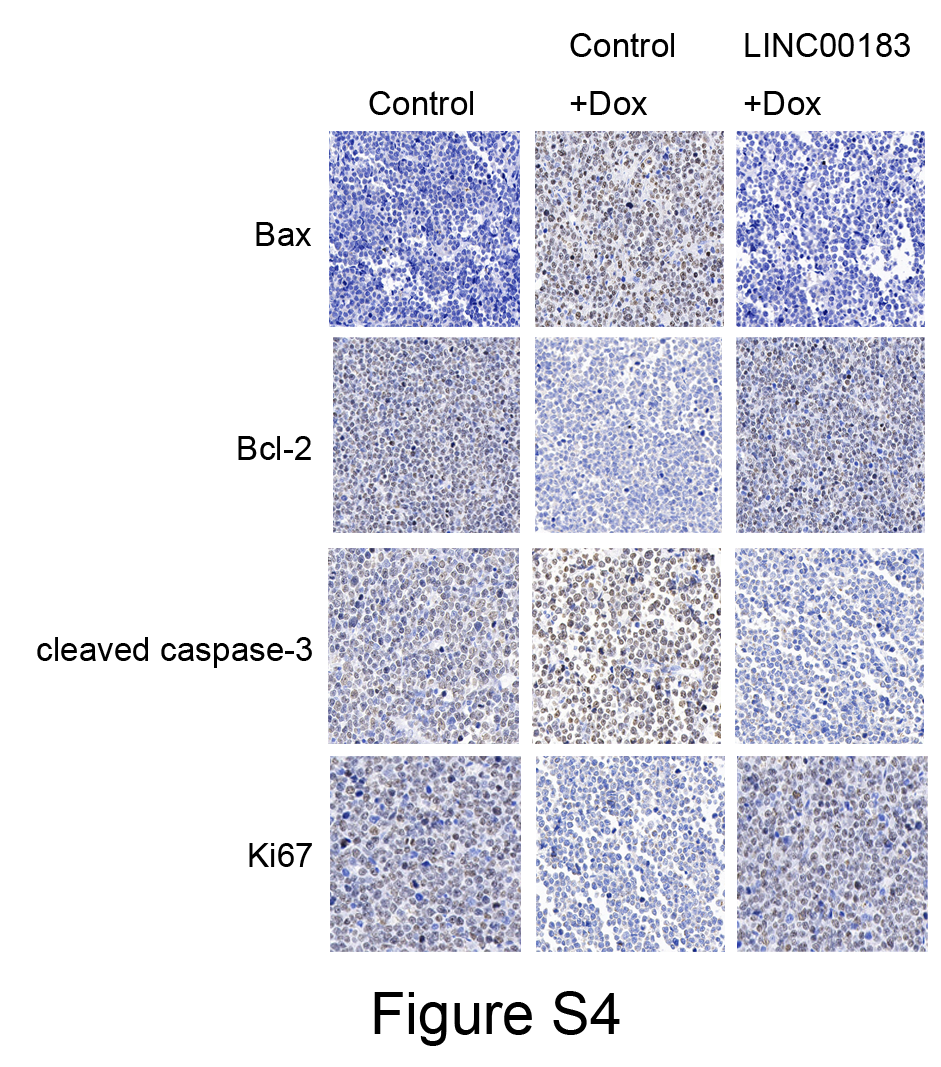

Supplement: Supplementary file 6 — Supplementary Material 6 [file 13046_2023_2670_MOESM6_ESM.tif]

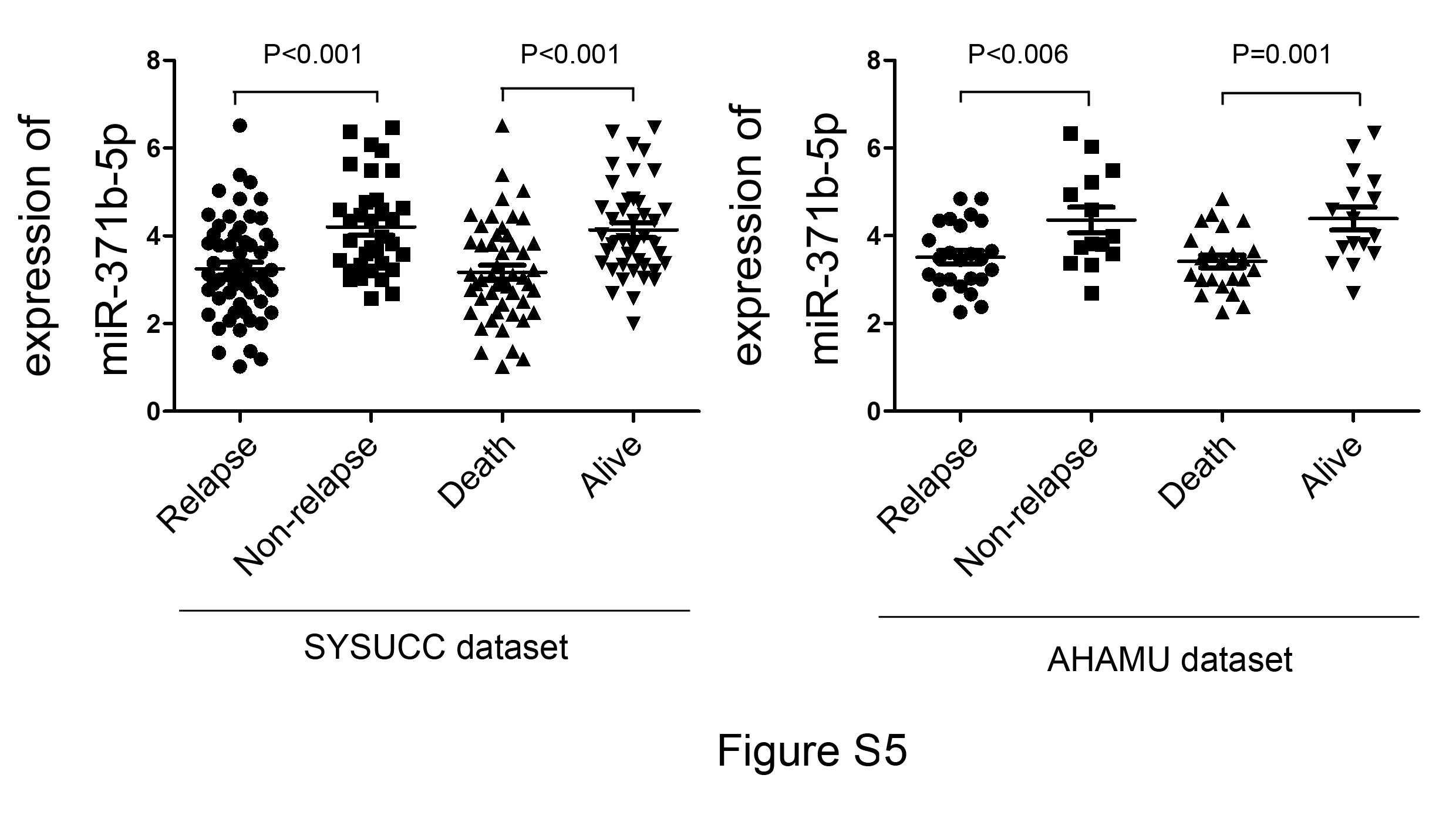

Supplement: Supplementary file 7 — Supplementary Material 7 [file 13046_2023_2670_MOESM7_ESM.tif]

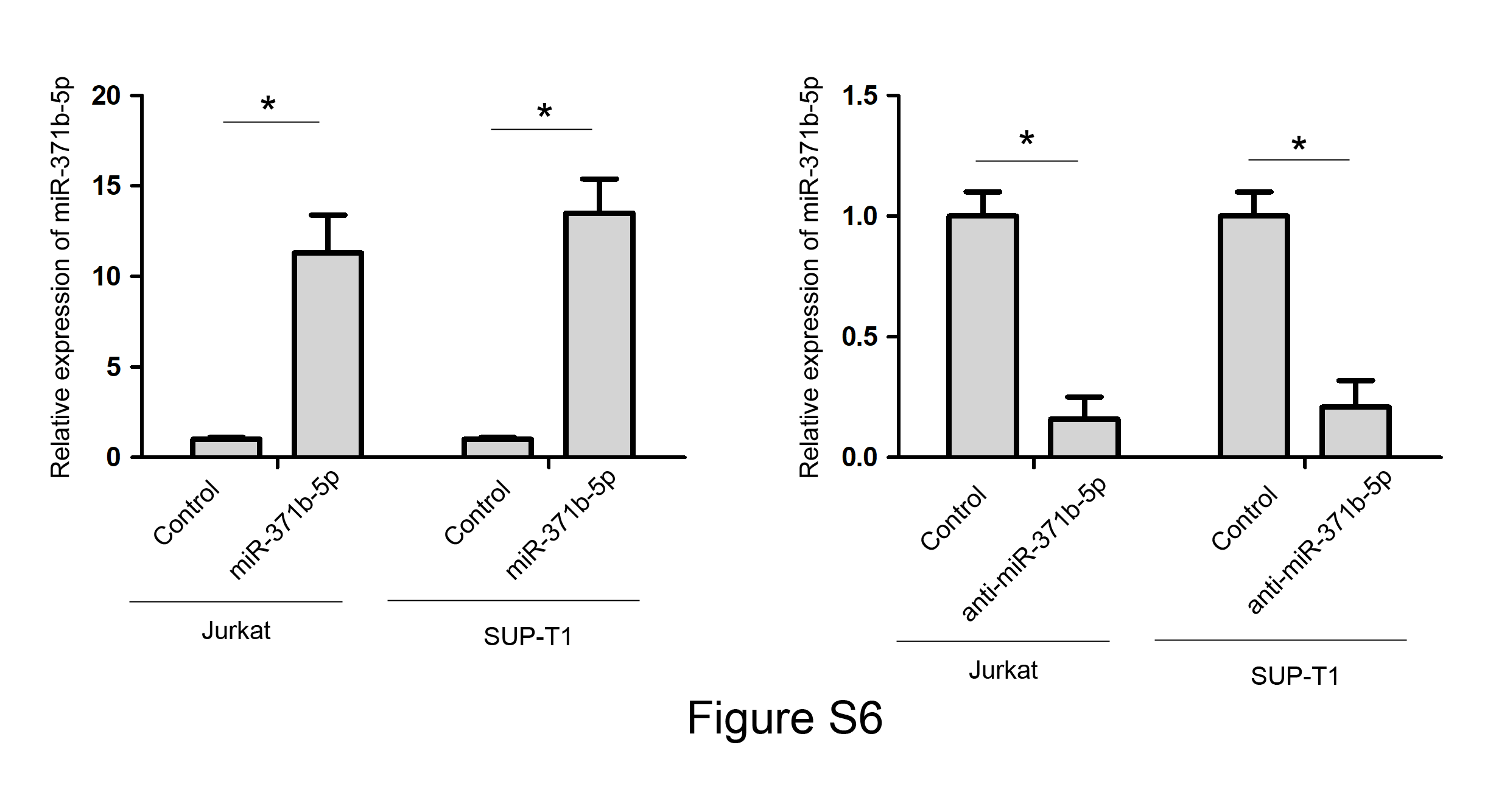

Supplement: Supplementary file 8 — Supplementary Material 8 [file 13046_2023_2670_MOESM8_ESM.tif]

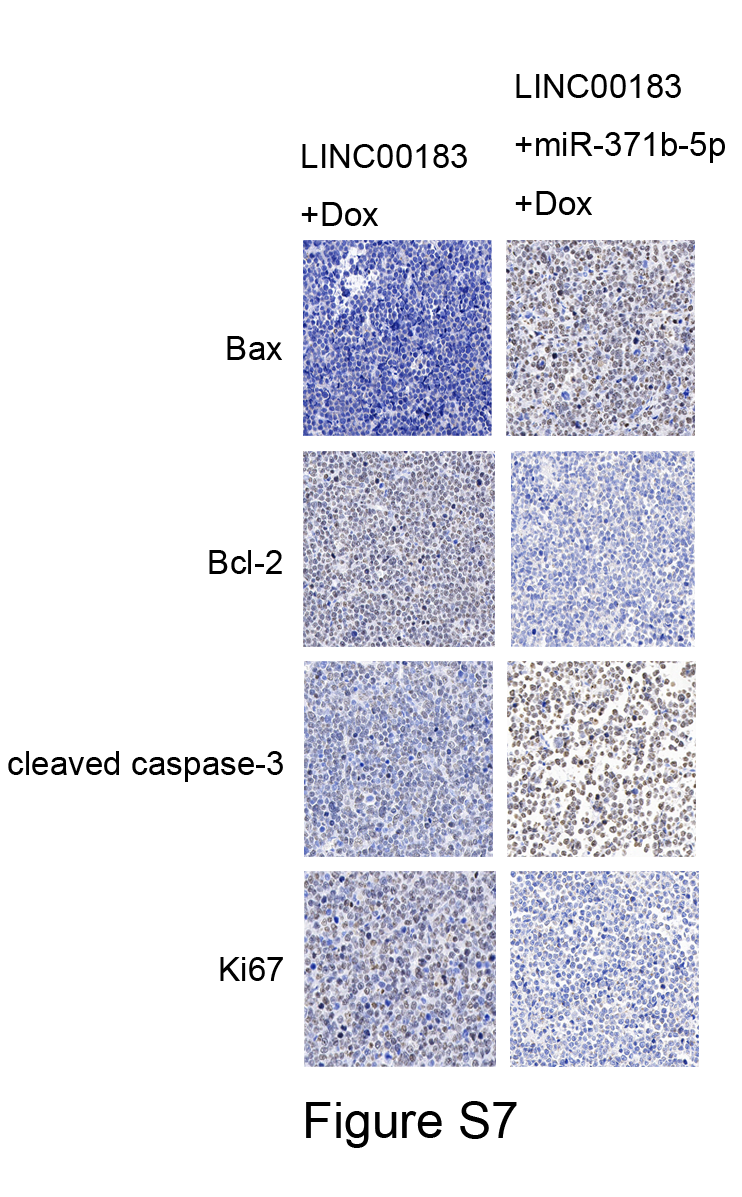

Supplement: Supplementary file 9 — Supplementary Material 9 [file 13046_2023_2670_MOESM9_ESM.tif]

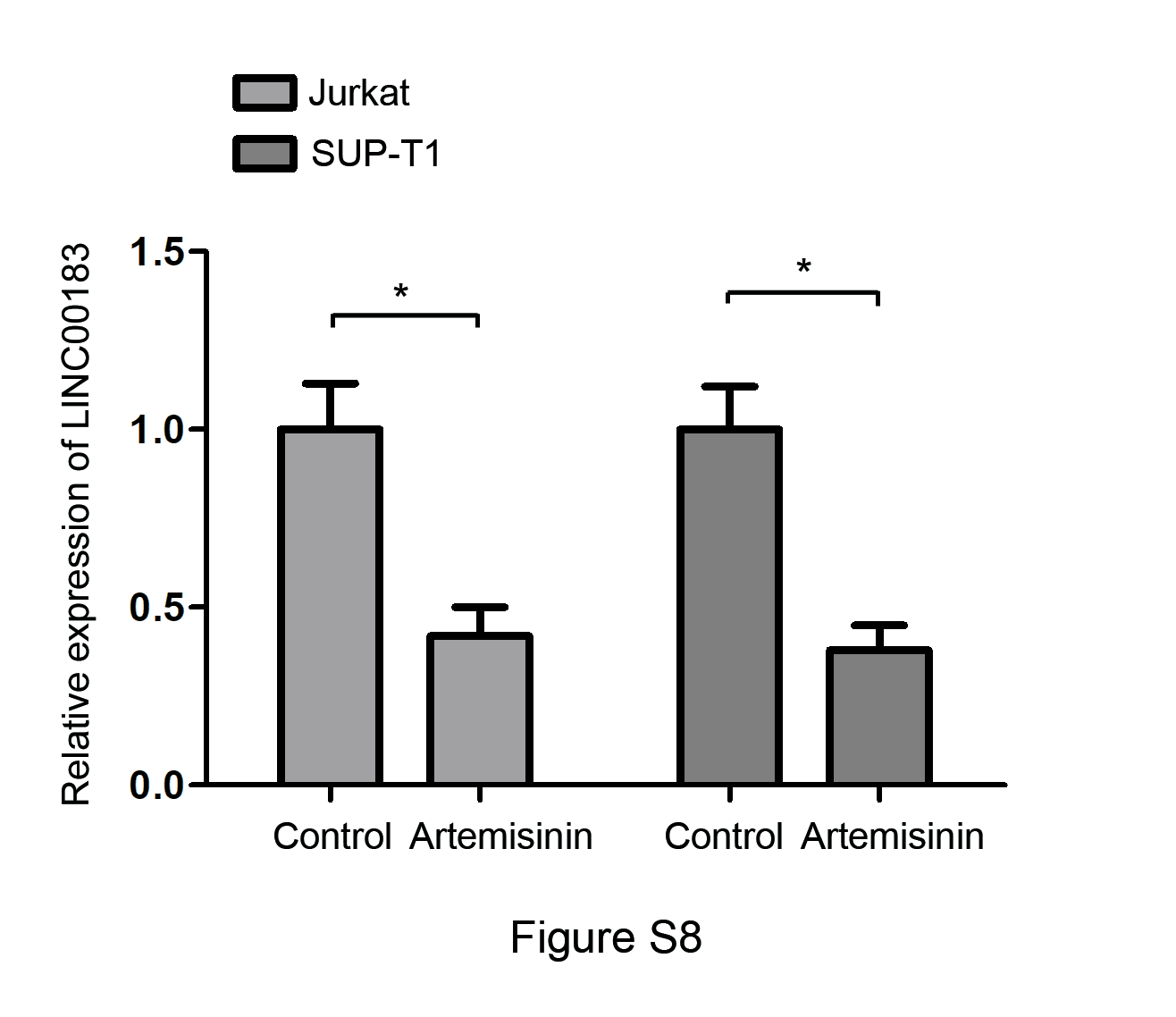

Supplement: Supplementary file 10 — Supplementary Material 10 [file 13046_2023_2670_MOESM10_ESM.tif]

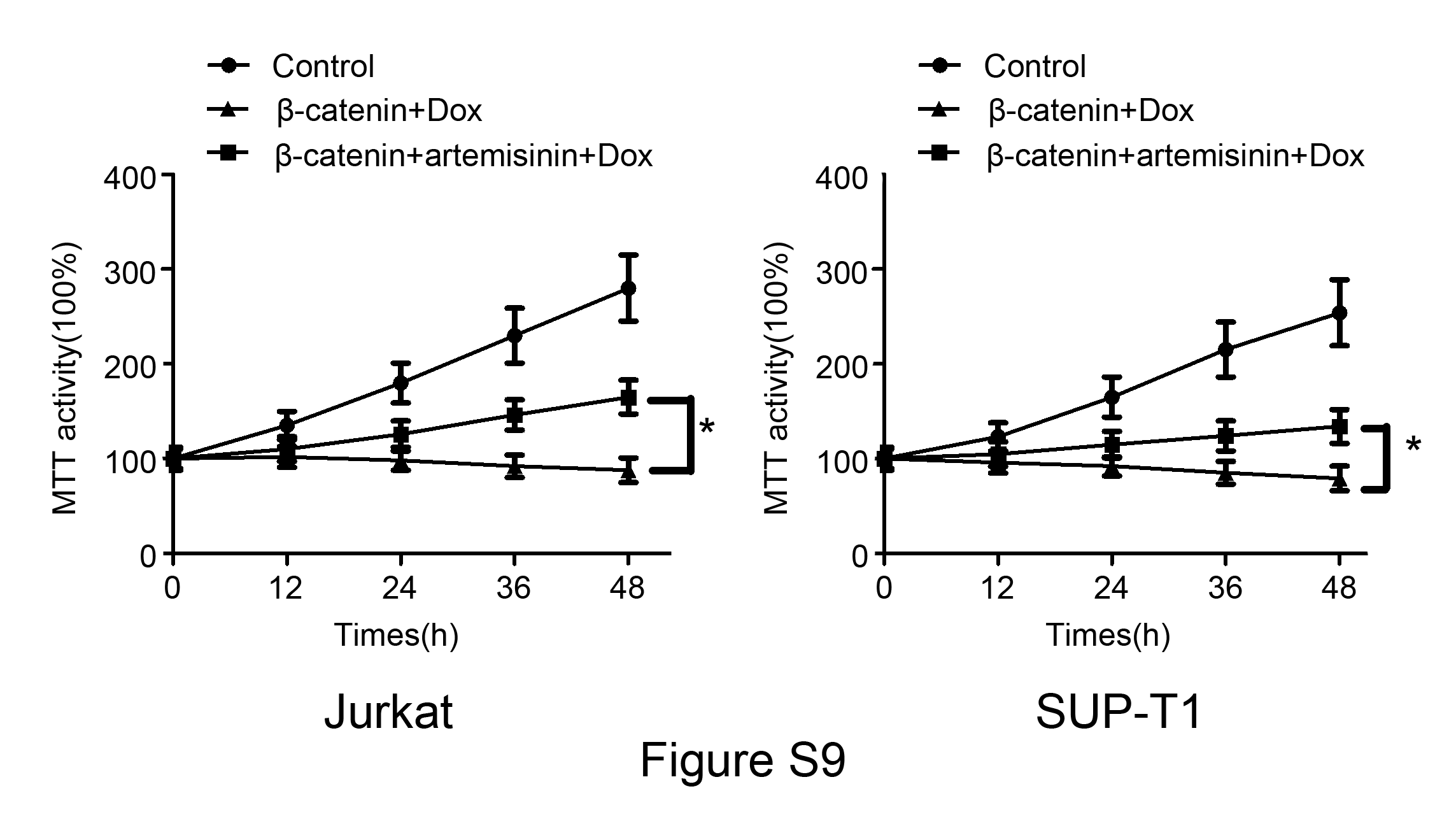

Supplement: Supplementary file 11 — Supplementary Material 11 [file 13046_2023_2670_MOESM11_ESM.tif]
