# Supplementary material for: The β-catenin-LINC00183-miR-371b-5p-Smad2/LEF1 axis promotes adult T-cell lymphoblastic lymphoma progression and chemoresistance
Source: J Exp Clin Cancer Res. 2023 Apr 28;42:105. doi: 10.1186/s13046-023-02670-9 (PMC10141948; doi:10.1186/s13046-023-02670-9)
Supplement: Supplementary file 12 — Supplementary Material 12 [file 13046_2023_2670_MOESM12_ESM.docx]

**Supplementary Figure legend**

**Figure S1. The different expression of LINC00183 between relapse and non-relapse, death and alive in SYSUCC dataset and AHAMU dataset.**

**Figure S2. The relative expression of LINC00183 in normal T cells and T-LBL cells transfected by LINC00183 and shLINC00183.** LINC00183, ectopic LINC00183 expression in T-LBL cells. shLINC00183, T-LBL cells were transfected by shRNA targeting LINC00183. *, *P*<0.05.

**Figure S3. The IC50 for Dox for the Jurkat and SUP-T1 cells with LIN00183 expression manipulation and mouse. A**. IC50 for Dox for the Jurkat; **B.** IC50 for Dox for the Jurkat-LINC00183; **C.** SUP-T1 IC50 for Dox for the SUP-T1; **D.** SUP-T1 IC50 for Dox for the SUP-T1-LINC00183; **E**. IC50 for mouse. LINC00183, ectopic LINC00183 expression in T-LBL cells. Dox, doxorubicin.

**Figure S4. The immunohistochemical staining assays of Bcl-2, Bax, cleaved-caspase-3 and Ki67 expression in mouse xenografts bearing SUP-T1 and SUP-T1-LINC00183 cells treated with doxorubicin.** SUP-T1-LINC00183, SUP-T1 cells transfected with LINC00183. Dox, doxorubicin.

**Figure S5. The relative expression of miR-371b-5p in SYSUCC and AHAMU dataset.**

**Figure S6. The relative expression of miR-371b-5p in T-LBL cells transfected by miR-371b-5p and anti-miR-371b-5p.** *, *P*<0.05.

**Figure S7. The immunohistochemical staining assays of Bcl-2, Bax, cleaved-caspase-3 and Ki67 expression in mouse xenografts bearing SUP-T1-LINC00183 and SUP-T1-LINC00183-miR-371b-5p cells treated with doxorubicin.** SUP-T1-LINC00183, SUP-T1 cells transfected with LINC00183. SUP-T1- LINC00183-miR-371b-5p, SUP-T1 cells transfected with LINC00183 and miR-371b-5p. Dox, doxorubicin.

**Figure S8. The relative expression of LINC00183 in T-LBL cells treated with artemisinin.** LINC00183, ectopic LINC00183 expression in T-LBL cells. *, *P*<0.05.

**Figure S9. The MTT assay of T-LBL cells treated with artemisinin.** β-catenin, T-LBL cells were transfected with β-catenin. Dox, doxorubicin. *, *P*<0.05.
